# Does daily co-administration of letrozole and gonadotropins during ovarian stimulation improve IVF outcome?

**DOI:** 10.1186/s12958-017-0288-8

**Published:** 2017-08-30

**Authors:** Jigal Haas, Rawad Bassil, Jim Meriano, Nivin Samara, Eran Barzilay, Noa Gonen, Robert F. Casper

**Affiliations:** 1 Division of Reproductive Sciences, University of Toronto, Lunenfeld-Tanenbaum Research Institute, Mount Sinai Hospital, Toronto, ON Canada; 2 TRIO fertility partners, 655 Bay St, Toronto, ON M5G 2K4 Canada; 3 Chaim Sheba medical Center, Tel Hashomer, affiliated with the Sackler Faculty of Medicine, Tel Aviv University, Tel Aviv, Israel

**Keywords:** Letrozole, Androgen, Aromatase inhibitor

## Abstract

**Background:**

For the last year we have been treating normal responders with gonadotropins and letrozole during the whole stimulation in order to improve response to FSH by increasing the intrafollicular androgen concentration, and to reduce circulating estrogen concentrations. The aim of this study was to compare the IVF outcome of normal responders treated with letrozole and gonadotropins during ovarian stimulation with patients treated with gonadotropins only.

**Methods:**

A single centre retrospective cohort study of 174 patients (87 in each group).

**Results:**

The age of the patients was comparable between the groups. Estradiol levels were significantly higher in the control group (6760 pmol/L vs. 2420 pmol/L respectively, *p* < 0.01), and the number of follicles ≥15 mm at the trigger day was significantly lower in the control group (7.9 vs. 10, *p* = 0.02). The number of retrieved oocytes (10 vs. 14.5, *p* < 0.01), MII oocytes (7.9 vs. 11.2, *p* < 0.01) and blastocysts (2.7 vs. 4.0, *p* = 0.02) was significantly higher in the study group. We found no significant differences in the cumulative pregnancy outcome between the two groups (65.2% vs 58.3% p = NS).

**Conclusions:**

We conclude that co-treatment with letrozole improves the IVF outcome in normal responders in terms of increased number of blastocysts obtained without increasing the pregnancy rate or the risk of OHSS.

## Background

Previous studies have shown that androgens, in addition to serving as precursors for ovarian estrogen synthesis, also have a fundamental role in primate ovarian follicular development by augmentation of FSH receptor expression on granulosa cells [1].

Pre-treatment with transdermal testosterone was shown to improve the ovarian sensitivity to FSH and follicular response to gonadotrophin treatment in low-responder IVF patients [2] and resulted in an increase in the number of cumulus oocyte complexes retrieved, as well as clinical pregnancy and live birth rates [3].

Previous work by our group has shown that an aromatase inhibitor improves ovarian response to FSH in patients undergoing ovulation induction and intrauterine insemination [4]. Later, Lazer et al. [5] in a retrospective study, compared poor responders treated with letrozole and low dose gonadotropins vs. poor responders treated with only high dose gonadotropins during IVF cycles. They found a higher clinical pregnancy rate and a higher live birth rate in the group of patients treated with letrozole and low dose gonadotropins. Garcia-Velasco et al. [6] also found improved IVF cycle outcome in poor responder patients treated with high dose gonadotropins and letrozole compared to patients treated only with high dose gonadotropins. They also documented a significant increase in follicular fluid testosterone and androstenedione with letrozole administration for 5 days in the early follicular phase during IVF ovarian stimulation (6).

Considering the profound effect intraovarian androgens may have on the early follicular growth, we have been treating normal responders for the last year with gonadotropins and letrozole during the whole stimulation in order to increase the intrafollicular androgen concentration and to reduce circulating estrogen concentrations. This protocol is used routinely for ovarian stimulation in breast cancer patients and has been shown to have no adverse effect on the number of oocytes retrieved. However, the fertilization rate and embryo development rate of those oocytes is not always known [7, 8].

The aim of this study was to compare the IVF outcome of normal responders without breast cancer treated with letrozole and gonadotropins during ovarian stimulation compared to patients treated with gonadotropins only.

## Methods

This was a single center, retrospective cohort study of 174 IVF cycles between March 2016 and March 2017. The study was approved by the Research Ethics Board at Mount Sinai Hospital in Toronto.

Inclusion criteria were: Patients undergoing a fresh IVF stimulation cycle at age ≤ 42, with antral follicular count ≥4. The study group included patients on a GnRH antagonist protocol treated with daily letrozole 5 mg together with gonadotropins, from the first day of ovarian stimulation until the trigger day.

For each patient in the study group we matched a patient in the control group that was treated during the same week, at the same age ± 2 years, with the same infertility diagnosis, with less than three previous IVF cycles, FSH levels under ten and the same gonadotropin dose ±75 IU, on a GnRH antagonist protocol, treated with gonadotropins without letrozole during the stimulation.

The decision whether to co-treat with letrozole was made by the treating physicians independently between March 2016 and March 2017.

### Stimulation protocols

Gonadotropin treatment (with or without letrozole) was initiated on the 3rd day of menses with the use of recombinant FSH (Gonal F, EMD Serono; or Puregon, Merck). Once the leading follicle had reached a size of 13 mm, or E2 levels exceeded 1200 pmol/L, co-treatment with GnRH antagonist 0.25 mg/day (Cetrotide, Serono or Orgalutran, Merck) and recombinant LH (Luveris, Serono) or highly purified human menopausal gonadotropin (Menopur, Ferring) was commenced. Follicle growth and hormone levels were serially monitored by ultrasound and blood tests until the dominant follicles reached an average diameter of 18–20 mm. At that point human chorionic gonadotropin (10,000 IU Pregnyl; Merck, Kirkland, Quebec) and GnRH agonist (0.5 mg Suprefact; Sanofi-Aventis, Canada) were administered subcutaneously to trigger ovulation. Patients at high risk to develop OHSS (previous history of OHSS or patients with ≥20 follicles at size ≥10 mm at the trigger day) were triggered only with GnRH agonist. Thirty-six hours later oocyte retrieval was performed under transvaginal guided ultrasound and needle aspiration. The embryos were transferred during a fresh or frozen cycle, as decided independently by the treating physician.

All blastocyst were evaluated by an experienced embryologist using the grading system proposed by Gardner [9].

The frozen embryos were thawed and transferred during modified natural cycles or hormonally substituted frozen embryo transfer (FET) cycles.

### Frozen cycle endometrial preparation

#### Artificial hormone replacement

Patients started on day 2–3 of the cycle with oral administration of 2 mg of estradiol (Estrace) twice daily for endometrial preparation, which was increased by a step-up protocol to 8 mg/d. An ultrasound endometrial assessment performed about 10 days later assessed the lining as ready for the ET procedure when the endometrial thickness was ≥7.0 mm. If not adequate, endometrial estrogen priming continued and ultrasound assessment was undertaken to confirm further endometrial thickening. Participants commenced luteal support via vaginal administration of progesterone suppositories 200 mg three times daily according to the proposed day of embryo thawing and transfer. The embryos were thawed on day 6 of progesterone and transferred after 2–4 h.

#### Natural cycles

Following spontaneous menstruation, patients were monitored by serial ultrasound for endometrial thickness and follicular development, and blood was drawn for measurement of serum LH, estradiol and progesterone levels, until a surge in LH was observed defined as an increase in serum LH of 200% of the baseline value). The first day of the LH surge was considered as corresponding to 1 day prior to ovulation. On the following day, progesterone suppositories 200 mg three times daily were started. The embryos were thawed on day 6 of progesterone and transferred after 2–4 h.

Vitrification was performed using the Irvine Scientific (Irvine Scientific Santa Ana Ca, USA) “Freeze Kit” (Cat.#90,133-SO) with HSV straws.

Clinical pregnancy was defined as visualization of a gestational sac, while ongoing pregnancy necessitated the visualization of fetal cardiac activity past 10 weeks gestation on transvaginal ultrasound.

### Statistics

Comparison of continuous variables between the two groups was conducted using Student T test and Mann Whitney test as appropriate. Chi-square test was used for comparison of categorical variables. Significance was accepted at *P* < 0.05. Statistical analyses were conducted using the IBM Statistical Package for the Social Sciences (IBM SPSS v.20; IBM Corporation Inc., Armonk, NY, USA).

## Results

One hundred seventy four patients were included in the study, 87 patients in each group. The infertility diagnosis, FSH levels, previous number of IVF treatments, age of the patients and the total dose of gonadotropins were comparable between the two groups (Table 1). The length of stimulation was significantly shorter in the letrozole group compared to the control group (10.2 days vs 11.1 days, *p* < 0.01). Estradiol levels were significantly higher in the control group (6760 pmol/L vs. 2420 pmol/L, *p* < 0.01) and the number of follicles ≥15 mm at the trigger day was significantly lower in the control group compared to the study group (7.9 vs. 10, *p* = 0.02).The number of eggs/number of follicles ≥ 15 mm was similar in both groups (134% vs. 142%, p = NS).

**Table 1:** Characteristics of the IVF cycles for patients co-treated with letrozole compared to the control group

	Control group	Letrozole	*P*
*N*	87	87	
Age (years)	37.0 ± 3.8	36.5 ± 4.1	NS
Length of stimulation (days)	11.1 ± 2.1	10.2 ± 1.7	<0.01
Total dose of gonadotropins (IU)	3012 ± 1115	3316 ± 1037	NS
Follicles >15 mm at the trigger day	7.9 ± 5.3	10 ± 5.8	0.02
E2(pmol/L)	6760 ± 4792	2420 ± 1923	<0.01

**Table 2:** Comparing the IVF outcome between patients co-treated with letrozole and control group

	Control group	Letrozole	*p*
Eggs(*n*)	10 ± 6.0	14.5 ± 9.3	<0.01
Eggs/follicles > 15 mm	136 ± 58%	142 ± 57%	NS
MII(*n*)	7.9 ± 5.3	11.2 ± 7.8	<0.01
Blastocysts(*n*)	2.7 ± 2	4.0 ± 3.7	0.02
Pregnancy rate (Fresh + frozen)^a^	15/23 (65.2%)	14/24 (58.3%)	NS
Pregnancy rate- fresh transfers	8/30 (26.6%)	9/24 (37.5%)	NS
Cases of OHSS	0	0	---

^a^- only completed cycles were included in the analysis

The number of retrieved oocytes (10 vs. 14.5, *p* < 0.01), MII oocytes (7.9 vs. 11.2, *p* < 0.01) and blastocysts (2.7 vs. 4.0, *p* = 0.02) were significantly higher in the letrozole group (Table 2).

We found no significant differences in the ongoing cumulative pregnancy outcome (fresh + frozen cycles) (65.2% vs 58.3% p = NS) or in the ongoing pregnancy rate from the fresh cycles (26.6% vs 37.5% p = NS). Many of the cycles did not proceed to fresh transfer and the final cumulative pregnancy rate after all frozen embryo transfers is not yet available (many patients were freezing embryos for optional usage in the future). There were no cases of ovarian hyperstimulation (OHSS) in either group.

## Discussion

This study reports the use of concomitant letrozole and gonadotropin ovarian stimulation in normal responder patients without breast cancer. The IVF cycles of the patients treated with letrozole resulted in an increased number of mature eggs and more blastocysts without any cases of OHSS.

Aromatase is the rate-limiting enzyme in oestrogen biosynthesis, and inhibition of its activity reduces oestrogen blood levels and negative feedback on gonadotrophin secretion. Letrozole is the dominant aromatase inhibitor in use in infertility treatment for different subfertility/infertility indications. Letrozole is used “off label” in North America and also in many other countries around the world. The warning letter published by the original manufacturer is still the main obstacle to wider acceptance [10]. In the last few years, many studies have been published describing favourable results with use of letrozole in reproductive technologies with no short or long-term side effects [11–13]. We believe, in the light of the accumulating clinical research evidence, that letrozole is safe for use in assisted reproduction.

Recently, more and more studies demonstrated the potential beneficial use of letrozole in in-vitro fertilisation (IVF) cycles especially in breast cancer patients going through fertility preservation treatment [7, 8, 14, 15].

Quinn et al. compared the outcomes of women with breast cancer undergoing elective fertility preservation treated with and without letrozole. Patients treated with letrozole had more follicles ≥13 mm on the day of trigger injection and higher numbers of total oocytes retrieved compared to the elective fertility preservation group, but there was no difference in the number of MII oocytes or the mature oocyte yield [8].

In contrast to the previous breast cancer studies, the patients in the present study had their oocytes fertilized. Therefore, we have information regarding the fertilization rate and number of blastocysts per cycle. We found not only an increased number of mature oocytes, but also significantly more blastocysts in the group of women co-treated with letrozole.

There are a few potential advantages to co treating with letrozole during the IVF stimulation cycle: 1) Recruiting more follicles and increasing the number of retrieved oocytes, 2) Decreased estrogen concentrations in serum (and likely follicular fluid) possibly lowering the risk of OHSS. 3) Lower levels of E2 during the stimulation which might improve placental implantation and invasion following embryo transfers, and 4) a decrease in the risk of thromboembolic events related also to lower serum estradiol concentrations.

Whether letrozole might reduce the risk of OHSS by reducing oestrogen levels is controversial. A study in a rat model of OHSS demonstrated that treatment with letrozole reduced vascular endothelial growth factor (VEGF) and increased pigment epithelium derived factor (PEDF). VEGF has been identified as one of the prime causative factors in OHSS while PEDF has been shown to decrease anti-angiogenic activity of VEGF. The combined result should lead to a decrease in the incidence of OHSS [16].

He et al. demonstrated a dose dependent decrease in the levels of VEGF with increasing doses of letrozole administered in the luteal phase [17]. The findings reported above suggest that letrozole could decrease the risk of OHSS although it is not clear if the effect on VEGF and PEDF secretion is a direct action of letrozole or an indirect effect through a reduction in estradiol levels.

A randomized controlled study which aimed to compare the efficacy of letrozole to aspirin in primary prevention of early ovarian hyperstimulation syndrome showed a lower incidence of OHSS in women receiving letrozole compared with aspirin [14]. The lower incidence of OHSS was not correlated to lower levels of VEGF in the serum. The authors hypothesized that the mechanism might be independent of VEGF but rather due to the induction of a luteolytic effect and an estradiol decline which reduced the risk of early-onset OHSS.

Epidemiological studies suggest that increased levels of estradiol resulting from the oral contraceptive pill, hormone replacement therapy [18] or during pregnancy [19] is associated with increased risk for thromboembolic events. During IVF treatment, patients developing OHSS with hemoconcentration and high levels of E2 are at an increased risk to suffer from thromboembolic events [20]. We hypothesize that co-treatment with letrozole, resulting in lower levels of oestrogen, will decrease the risk of developing a thromboembolic event.

Integrin expression, a marker of endometrial receptivity, has been shown to be reduced in women with IVF failure [21] and in women with endometriosis [22]. Miller et al. aimed to examine the effect of letrozole on integrin expression as a marker of endometrial receptivity. They found that a lack of endometrial integrin expression was associated with a poor prognosis for IVF and that the results were improved with letrozole co-treatment [23]. Unfortunately our study was underpowered to detect any differences in pregnancy outcome during fresh transfers due to the low number of fresh transfers at our clinic (less than 30%). However, superphysiologic levels of circulating estradiol may lead to decreased implantation rate [24] and to deficient placentation leading to pregnancy complications such as SGA and IUGR which seem to be avoided by FET when estrogen levels in the circulation are more physiologic [25, 26]. The estrogen levels in the group receiving letrozole in the present study were close to the physiologic range despite a mean number of 14 oocytes retrieved.

There are a few limitations to our study. The main limitation is the retrospective study design and the physician choice of allocation to either group, both of which would introduce bias. There are other factors that can influence IVF outcome such as the physician and the embryologist performing the retrieval/transfer and those factors were not controlled for in the study. Another limitation is the lack of pregnancy outcome in some of the patients. Many of the patients and their physicians opted for frozen embryo transfer instead of fresh transfer, especially in cases of overstimulation, and a cumulative pregnancy outcome for each group is not available.

Although the results of this study are promising further prospective studies will be needed to confirm that patients co-treated with letrozole have a better clinical outcome.

## Conclusion

We observed, in a retrospective study, that co-treatment with letrozole during the ovarian stimulation improves the IVF outcome in normal responders in terms of increased number of blastocysts obtained without increasing the pregnancy rate and the risk of OHSS.

## Abbreviations

ET: Embryo transfer
FET: Frozen embryo transfer
FSH: Follicle stimulating hormone
IVF: In vitro fertilization
OHSS: Ovarian hyperstimulation
